# The Safety and Efficacy of an Enzyme Combination in Managing Knee Osteoarthritis Pain in Adults: A Randomized, Double-Blind, Placebo-Controlled Trial

**DOI:** 10.1155/2015/251521

**Published:** 2015-01-31

**Authors:** Wolfgang W. Bolten, Michael J. Glade, Sonja Raum, Barry W. Ritz

**Affiliations:** ^1^Klaus-Miehlke Klinik, Leibnizstraße 23, 65191 Wiesbaden, Germany; ^2^The Nutrition Doctor, 8612 Kedvale Avenue, Skokie, IL 60076, USA; ^3^Mucos Pharma GmbH & Co. KG, Miraustraße 17, 13509 Berlin, Germany; ^4^Atrium Innovations, 4 Hillman Drive, Suite 190, Chadds Ford, PA 19317, USA

## Abstract

This randomized, double-blind, placebo-controlled, and comparator-controlled trial evaluated the safety and efficacy of an enzyme combination, as Wobenzym, in adults with moderate-to-severe osteoarthritis (OA) of the knee. Adults (*n* = 150) received Wobenzym, diclofenac (a nonsteroidal anti-inflammatory drug, NSAID), or placebo for 12 weeks. Improvement in pain scores (Lequesne Functional Index) did not differ between subjects treated with Wobenzym or diclofenac, and both treatment groups improved compared to placebo (*P* < 0.05). Reduction in total WOMAC scores (secondary outcome measure) did not differ between Wobenzym and diclofenac, although only diclofenac emerged as different from placebo (*P* < 0.05). The median number of rescue medication (paracetamol) tablets consumed was less in the Wobenzym group compared to placebo (*P* < 0.05), while there was no difference between diclofenac and placebo. Adverse events were similar in frequency in Wobenzym and placebo groups (7.2% and 9.1% of subjects, resp.) and higher in diclofenac group (15.6%). Wobenzym is comparable to the NSAID diclofenac in relieving pain and increasing function in adults with moderate-to-severe painful knee OA and reduces reliance on analgesic medication. Wobenzym is associated with fewer adverse events and, therefore, may be appropriate for long-term use.

## 1. Introduction

Osteoarthritis (OA), known as degenerative joint disease, is characterized by gradual loss of joint cartilage and intermittent or persistent local inflammatory processes. The hallmark signs of this progressive disorder are limitations in joint movement and pain. OA is the most common form of arthritis affecting nearly 14% of adults aged 25 to 65 and nearly 34% of adults over the age of 65 [1]. The most common site of osteoarthritis in individuals 40 years and older is the knee [2].

The signature pathologic feature of osteoarthritis is the progressive net loss of hyaline cartilage from within the joint, driven by the chondrocytic responses to aberrant loading of mechanical forces within the joint “mechanopathology,” with concomitant inflammatory responses to the degeneration of joint structures [3–5]. Nonsteroidal anti-inflammatory drugs (NSAIDs) are effective in reducing inflammation and pain but are associated with significant side effects that can interfere with therapeutic success, especially with long-term treatment [6].

Wobenzym is an orally administered combination of proteolytic enzymes and bioflavonoid. In combination, these active principles have produced beneficial outcomes in subjects with osteoarthritis of the knee and hip which were equivalent to those produced by the NSAID diclofenac [7–11]. However, these studies lacked a placebo control group.

In this current randomized, double-blind clinical trial we compared the effectiveness of an enzyme combination, as Wobenzym, with the effectiveness of the NSAID diclofenac and placebo. Outcome measures included amelioration of pain, improvement of function, and type and frequency of side effects.

## 2. Methods

### 2.1. Data Integrity

The conduct of the experiment and the integrity of the collected data were monitored through oversight provided by Monitor Zentrale Deutschland (Sauerlach, Germany). The accuracy of the collected data was overseen by Clinical Research Facilities International (Schaijk, Netherlands). The trial was reviewed and approved by an independent ethics committee and was performed according to the principles defined in the current edition of the Declaration of Helsinki, according to German Drug Law (AMG), and according to Good Clinical Practice (GCP). This trial was registered with ClinicalTrials.gov on March 13, 2014 (identifier NCT02088411).

### 2.2. Subjects

Middle-aged and older adult volunteers with moderate-to-severe knee osteoarthritis were enrolled in the study after satisfying all inclusion and exclusion criteria. Briefly, inclusion required the age of 40–80, a score on Lequesne Functional Index of 10–14 (the maximum possible index score is 24), WOMAC-A pain subscale score of ≥25 (scores range from 0 to 100), and arthritis of the knee confirmed by conventional radiography or tomography and swelling of the affected knee upon physical examination. Subjects were excluded based on a history of knee trauma, joint infection, joint surgery, or intra-articular injection (viscotherapy). Subjects with gastrointestinal diseases or with pharmacotherapy with corticosteroids, COX-II inhibitors, or glucosamine/chondroitin were excluded, as well as patients with known sensitivity to paracetamol, NSAIDs, or oral enzymes. Written informed consent was obtained from each patient prior to study enrollment following an oral and written explanation about the aim and the potential risks of the study.

### 2.3. Interventions

Subjects were recruited, screened, evaluated for eligibility, and enrolled into the study at clinical centers located throughout Germany (Bensheim, Berlin, Essen, Hamburg, Luneburg, Munchen, Norderstedt, Siegen, Stockach, Wiesbaden, and Wolfratshausen) and 2 centers in the Netherlands (Oos, Zwoll). Subjects were assigned randomly to receive three times daily either 1 tablet of diclofenac sodium (50 mg; Merckle GmbH, Blaubeuren, Germany) and 2 tablets of an indistinguishable placebo, or 2 tablets of Wobenzym (Mucos Pharma GmbH & Co. KG, Berlin, Germany) and 1 tablet of placebo, or 3 tablets of placebo. The resultant doses of active treatments were, therefore, Wobenzym 6 tablets (2 tablets, 3 times daily) or diclofenac 150 mg (1 tablet, 3 times daily). All interventions were identical in the total number of tablets and appearance. The random assignment of subjects to treatment groups was achieved through the use of a computer-generated randomization list in continuous recruitment order.

Wobenzym is an oral combination of natural compounds, including 288 mg trypsin (from porcine or bovine pancreas), 540 mg bromelain (from pineapples,* Ananas comosus*), and 600 mg rutoside trihydrate (rutin; from Japanese pagoda tree,* Sophora japonica*) per recommended daily dose. The tablets are enteric coated to prevent inactivation of the enzymes during gastric passage. The tablets must be consumed on an empty stomach, separate from meals.

### 2.4. Study Protocol

Each treatment was administered daily for 12 weeks. Compliance was determined by counting unconsumed tablets during examinations that were scheduled at 4, 8, and 12 weeks of the intervention. A minimum of 75% compliance was required. When necessary the subjects were allowed to self-medicate with the rescue medication paracetamol (acetaminophen, 500 mg per tablet) up to 2000 mg in a single 24-hour period. The use of rescue medication was not permitted 24 hours prior to each scheduled examination. The incidence of use and amount of paracetamol consumed were recorded in a patient diary. Other antirheumatic or analgesic therapies were prohibited, although continued use of daily low-dose (81 mg) aspirin for cardiovascular health was permitted. Self-administered exercise was allowed but the initiation of a new physical therapy regimen was prohibited.

### 2.5. Outcomes

The primary outcome was self-assessment of the affected knee joint by physician interview using the Lequesne Functional Index and reported as the total score. This index provides an estimate of the degree of pain associated with the affected joint, the maximum distance walked, and the activities of daily living [12]. Additional outcome criteria focused on self-assessments of pain and function utilizing the Western Ontario and McMaster Universities Index version 3.0 subscales for pain (WOMAC-A), joint stiffness (WOMAC-B), and physical joint function (WOMAC-C), as well as the total score after combining all three subscale scores [13]. In addition, selected indices of systemic inflammation (erythrocyte sedimentation rates at 1 and 2 hours and serum concentration of C-reactive protein) were evaluated [14]. These assessments were performed at baseline and 12 weeks.

The safety of each treatment was assessed by documenting the occurrence, nature, severity, and relevance of all adverse events, as well as potential adverse drug reactions that occurred during the course of the study, either recorded in patient diaries or communicated directly to study personnel. In addition, the following vital signs and blood chemistries were measured at baseline and week 12: resting systolic and diastolic blood pressure, heart rate, and body temperature; hematocrit; red and white blood cell count; thrombocyte count; fasting blood glucose concentration; fasting serum concentrations of total protein, albumin, urea-nitrogen, total bilirubin, creatinine, hemoglobin, calcium, sodium, potassium, and chloride; fasting serum activities of alkaline phosphatase, glutamic-oxaloacetic transaminase (SGOT; also known as aspartate aminotransferase; ASAT), glutamic/glutamate pyruvic transaminase (SGPT; also known as alanine aminotransferase; ALAT), and *γ*-glutamyl transpeptidase (*γ*-GT; GGT); and prothrombin times and partial prothrombin times.

### 2.6. Data Analysis and Statistics

In order to account for baseline variability among subjects, the primary and secondary pain and joint function data were converted into the change and the percentage change for each subject during the 12 weeks of the study. Because the number of subjects that completed the study was too small for the meaningful application of tests of normality, all observations, 12-week changes, and 12-week percentage changes were expressed as medians (and their 95% confidence intervals) and were compared using nonparametric statistical procedures. Each combination of variable and treatment group, including the 12-week changes, 12-week percent changes, and nontransformed blood data, was tested against the null hypotheses of “no change” or “no percent change” using the sign test (*α* = 0.05) (InStat GraphPad Software, La Jolla, CA, USA). The change and percent change in each of the primary and secondary pain and joint function outcome variables and in each index of systemic inflammation were determined using the 2-sided Kruskal-Wallis procedure (*α* = 0.05) [15]. When the null hypothesis was rejected for a particular outcome, three pairwise comparisons were performed using the 2-sided Mann-Whitney procedure for unpaired data (*α* = 0.05). The median number of paracetamol tablets consumed by the subjects in the three treatment groups was compared using this same 2-step nonparametric approach. Within each treatment group, the percentage of subjects who consumed any paracetamol was compared to the percentage of subjects who consumed no paracetamol using the *z*-test for equality of proportions [15].

## 3. Results

**Figure 1:**
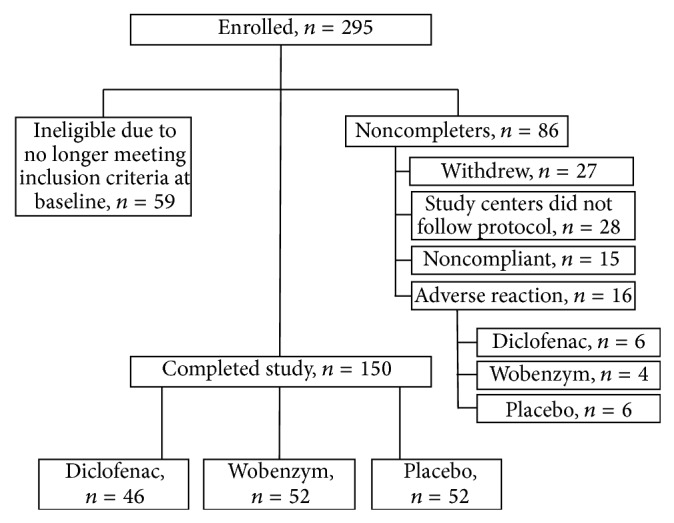


A total of 295 subjects were enrolled and randomly assigned to treatment groups. Of these, 150 completed the full 12 weeks of the trial according to protocol; 46 received diclofenac, 52 received Wobenzym, and 52 received placebo (Figure 1). According to protocol, 59 subjects were determined ineligible at baseline because they no longer met inclusion criteria for Lequesne and/or WOMAC pain scores. Of the other 86 subjects who did not complete the study, 27 withdrew voluntarily, 16 were discontinued due to adverse reactions, 28 were enrolled in two study centers that did not follow protocol, and 15 subjects were noncompliant. Reasons for withdrawal included “patient request” (4 subjects), perceived lack of efficacy (4 Wobenzym subjects, 4 diclofenac subjects, and 7 placebo subjects), loss to follow-up (3 subjects), and other protocol interruptions (5 subjects). Efficacy data were analyzed based on 150 completers. All enrolled subjects were included in the safety analysis.

### 3.1. Baseline Characteristics

Gender, age, body weight, height, body mass index, the number of months since the initial medical diagnosis of knee osteoarthritis, and the duration (in months) of active symptoms present upon enrollment did not differ between the treatment groups (Table 1).

### 3.2. Primary Outcomes

The primary outcome of affected knee joint function and joint-associated pain quantified using the Lequesne Functional Index improved in all three treatment groups during the 12 weeks of the study (Table 2). In the subjects treated with diclofenac or Wobenzym, both the absolute reduction in total score (median change: −4.0 points and −3.3 points, resp.) and the relative reduction in total score (median % change: −32.7% and −27.4%, resp.) were nearly twice those of the corresponding improvements in the subjects treated with placebo (−2.0 points, −16.4%) (*P* < 0.05). Absolute and relative reductions in pain did not differ between the diclofenac and Wobenzym treatment groups.

### 3.3. Secondary Outcomes

All three treatment groups exhibited similar improvements in absolute and relative scores on the WOMAC-A (joint pain) and WOMAC-B (joint stiffness) subscales (*P* < 0.05), and there were no significant differences between the groups (Table 3). Scores on the WOMAC-C subscale (joint function) and WOMAC total score were also reduced in all three groups (*P* < 0.05), but the relative reductions reached significance compared to placebo only among subjects treated with diclofenac (*P* < 0.05). There were no differences in WOMAC-C or WOMAC total score between the Wobenzym and diclofenac groups.

### 3.4. Paracetamol Consumption

The median number of paracetamol tablets consumed during the study was significantly less (*P* < 0.05) in the group treated with Wobenzym (0.5 tablets per subject in 12 weeks) compared to the placebo group (10.0 tablets per subject in 12 weeks), and the median number of paracetamol tablets consumed by subjects treated with diclofenac (4.0 tablets per subject in 12 weeks) did not differ from the other two groups (Table 4). Within each of the three treatment groups, the percentage of subjects who consumed any paracetamol (users) was not different from the percentage of subjects who consumed no paracetamol during the study.

### 3.5. Indices of Systemic Inflammation

Erythrocyte sedimentation rates and serum C-reactive protein concentrations were not affected in any group during the 12 weeks of the study (data not shown).

### 3.6. Safety

There were no relevant changes in vital signs or blood chemistries in any group (see supplemental data in the Supplementary Material available online at http://dx.doi.org/10.1155/2015/251521). During the study, 16 subjects were discontinued due to adverse events (4 Wobenzym subjects, 6 diclofenac subjects, and 6 placebo subjects). These were reported as mostly mild-to-moderate gastrointestinal events, such as nausea, reflux, and stomach pain (Table 5). In the entire enrolled study population, there were 32 recorded adverse events, primarily gastrointestinal, which were considered possibly related to the intervention. Among these, 7 were in the Wobenzym group (7.2%), 16 in the diclofenac group (15.6%), and 9 in the placebo group (9.1%). None of these events included ulcers or ulcer complications.

## 4. Discussion

The enzyme combination, as Wobenzym (2 tablets, three times daily, for 12 weeks), produced significant improvements in joint pain and function as measured by the Lequesne Functional Index in middle-aged and older adults with moderate-to-severe knee osteoarthritis. Self-assessed reductions in the degree of pain associated with the affected joint were recorded according to physician interview. There were significant improvements in the ability to walk for a distance and affected knee joint flexibility. The benefits of Wobenzym were comparable to those experienced with diclofenac (150 mg) and were significantly greater than the responses to placebo. The adverse event profile of Wobenzym was similar to placebo.

In addition, the use of rescue medication (number of tablets consumed), potentially an indirect measure of treatment efficacy, was reduced by 95% in the Wobenzym group compared to placebo (0.5 versus 10 tablets, resp.), while there was no significant difference in the use of rescue medication between the diclofenac and placebo groups or between the Wobenzym and diclofenac groups. There was no difference in the percentage of “paracetamol users” between the three groups. The use of rescue medication was associated with less response to treatment in all three groups, although this reached significance only in the diclofenac group (data not shown). Because (1) paracetamol rescue was required only when self-assessed pain exceeded a self-determined threshold of discomfort, (2) the same percentage of subjects resorted to pharmacologic rescue at least once in each group, and (3) the median number of paracetamol tablets consumed per subject was reduced in the enzyme group, these data suggest that use of Wobenzym was accompanied by fewer episodes of suprathreshold discomfort, a clinically relevant outcome observed during the 12 weeks of study.

Both the enzyme combination and diclofenac have been reported to exhibit anti-inflammatory activity [16–18]. OA is not associated with a measurable systemic but local inflammatory reaction. Accordingly the data in this study do not support an effect on markers of systemic inflammation. A downregulation of local inflammation in OA is accompanied by an improvement in clinical symptoms (reduction of swelling and pain), but not in lab parameters. Clinical observations suggest that local anti-inflammatory activity accompanied diclofenac and Wobenzym use and produced improved functionality. However, self-assessment of pain by WOMAC, a secondary outcome measure, and function (WOMAC-C subscale) differed between diclofenac and placebo but did not differ between Wobenzym and either placebo or diclofenac. The trend for improving in the Wobenzym group as seen in the WOMAC analysis was similar to the Lequesne outcome but did not reach statistical significance.

There is a large body of literature that explores the effectiveness and safety of the enzyme combination in managing joint pain [7–11, 16, 19–23]. Several studies also have reported enhanced mobility and recovery following sports injuries, including a faster return to training or competition [24–27]. However, the vast majority of these studies were comparator trials that did not include a placebo control. The value of such studies is limited because it is not possible to discern the additional benefits of the enzyme combination beyond the well-established influence of placebo on subjective measures of joint pain [28, 29]. When a placebo group is omitted from an experimental design and in clinical trials assessing the effectiveness of oral agents in OA, the “placebo effect” can be both statistically and clinically significant [30]. The current study included both a diclofenac group, as an active treatment comparator, and a placebo control. Previous studies demonstrating the effectiveness of Wobenzym in managing joint pain were primarily comparative studies establishing noninferiority compared to NSAID as standard of care [7–11]. The randomized, double-blind, placebo-controlled, and comparator-controlled trial design is, therefore, a major strength of this study. The close monitoring of rescue medication use is another strength of this study, as it provided an additional clinically relevant outcome measure.

While the multisite design is a strength of the study, the data obtained from the subjects from two clinical study sites were excluded from analysis because those study centers did not follow study protocol. This resulted in a smaller patient population sample available for analysis than originally anticipated. The large number of subject exclusions and resultant smaller sample size is the major limitation of the study and severely limits the ability to interpret these results. As such, the current study must be considered a reanalysis of the data.

The current study did not evaluate efficacy in the management of acute knee pain; therefore, no conclusions can be drawn regarding acute efficacy, such as time to onset. However, because of the progressive nature of this condition, finding a medication or supplement that can be used safely and effectively for the long term is a prudent clinical objective. The moderate length of this study does not conclusively demonstrate whether Wobenzym would remain effective beyond 12 weeks or whether Wobenzym would remain safer than diclofenac for longer-term use. However, the data suggest comparable efficacy and a better safety profile, as compared to NSAID, with repeated use over the study period of 12 weeks. While some data support the safe long-term use of the enzyme combination, such as up to 1–5 years, these studies are limited and concern subject populations not included here (rheumatoid arthritis, multiple sclerosis) [31–33]. Therefore, further controlled trials are needed to confirm whether Wobenzym may safely support knee joint function in an adult population over an extended period of time.

## 5. Conclusions

The enzyme combination Wobenzym may be as effective as the NSAID diclofenac in the management of chronic OA of the knee when administered for 12 weeks, with a similar safety profile to placebo, although further study is needed. Use of Wobenzym reduced the reliance on paracetamol (acetaminophen) for additional pain relief. Wobenzym may be a safe and effective option for the daily management of long-term joint pain.

## Supplementary Material

Subjects were administered diclofenac, Wobenzym®, or placebo for 12 weeks. No changes in vital signs or blood chemistries were observed.

## Figures and Tables

**Table 1:** Characteristics of subjects at baseline.

Characteristic	Treatment group		
	Diclofenac	Wobenzym	Placebo
Gender (% female)	76.1	67.3	76.9
Age (years)	63.5 (47–80)^1^	61 (47–80)	61.5 (44–81)
Body weight (kg)	79.5 (60–107)	79.5 (55–127)	78.0 (48–132)
Height (cm)	166 (150–183)	165 (147–193)	165 (150–185)
Body mass index	28.4 (21.5–44.3)	28.2 (21.7–37.9)	27.5 (16.2–44.4)

Months since initial diagnosis of knee osteoarthritis			
	55 (<1–261)	43.5 (1–397)	47.5 (2–417)

Duration of currently active symptoms (months)			
	3 (<1–245)	6 (<1–255)	5.5 (1–159)

^1^All values presented as medians (range).

**Table 2:** Lequesne Functional Index total scores.

	Median score		Median change	Median % change
	Baseline	Week 12		
Diclofenac	13.0^1^	8.3	−4.0^∗A^	−32.7^∗A^
	(12.0, 13.0)	(7.0, 10.0)	(−5.5, −2.0)	(−42.3, −22.8)

Wobenzym	12.0	9.0	−3.3^∗A^	−27.4^∗A^
	(11.5, 13.0)	(8.0, 10.0)	(−4.5, −2.0)	(−35.7, −16.7)

Placebo	12.5	9.8	−2.0^∗B^	−16.4^∗B^
	(12.0, 12.5)	(9.0, 11.5)	(−3.5, −0.5)	(−28.0, −4.8)

^1^All values presented as median (95% confidence interval).

^*^Significantly different from “no change” (*P* < 0.05).

^A,B^Treatment groups with different superscripts differ within the same column (*P* < 0.05).

**Table 3:** WOMAC subset and total scores.

	Median score		Median change	Median % change
	Baseline	Week 12		
WOMAC-A				
Diclofenac	29.0	15.0	−13.5^*^	−48.1^*^
	(28.0, 31.0)	(13.0, 17.0)	(−16.0, −11.0)	(−55.2, −40.0)
Wobenzym	31.0	19.0	−12.5^*^	−39.7^*^
	(30.0, 31.0)	(17.0, 23.0)	(−15.0, −10.0)	(−47.1, −28.0)
Placebo	30.0	21.0	−8.0^*^	−28.1^*^
	(28.0, 31.0)	(17.0, 25.0)	(−13.0, −5.0)	(−44.8, −16.7)
WOMAC-B				
Diclofenac	12.0	6.0	−4.0^*^	−43.7^*^
	(11.0, 13.0)	(5.0, 7.0)	(−6.0, −3.0)	(−57.1, −21.4)
Wobenzym	11.0	8.0	−4.0^*^	−33.3^*^
	(10.0, 12.0)	(6.0, 8.0)	(−4.0, −3.0)	(−41.7, −27.3)
Placebo	12.0	8.0	−3.0^*^	−26.2^*^
	(10.0, 13.0)	(6.0, 10.0)	(−4.0, −2.0)	(−35.7, −16.7)
WOMAC-C				
Diclofenac	100.0	53.0	−32.0^*^	−36.2^∗A^
	(93.0, 106.0)	(45.0, 64.0)	(−47.0, −20.0)	(−54.7, −24.0)
Wobenzym	101.5	70.0	−28.5^*^	−30.3^∗AB^
	(96.0, 108.0)	(65.0, 82.0)	(−37.0, −20.0)	(−39.4, −21.1)
Placebo	102.0	75.0	−19.0^*^	−19.7^∗B^
	(92.0, 107.0)	(66.0, 88.0)	(−27.0, −14.0)	(−34.3, −12.7)
WOMAC total				
Diclofenac	136.5	74.0	−50.0^*^	−38.3^∗A^
	(133.0, 146.0)	(62.0, 86.0)	(−73.0, −25.0)	(−54.4, −28.8)
Wobenzym	142.5	96.0	−46.5^*^	−35.5^∗AB^
	(136.0, 153.0)	(88.0, 114.0)	(−57.0, −34.0)	(−39.4, −21.1)
Placebo	144.5	101.5	−26.5^*^	−17.3^∗B^
	(129.0, 151.0)	(92.0, 123.0)	(−51.0, −22.0)	(−36.6, −12.5)

All values presented as medians (95% confidence interval).

^*^Significantly different from “no change” (*P* < 0.05).

^A,B^Treatment groups with different superscripts differ within the same column (*P* < 0.05).

**Table 4:** Paracetamol (acetaminophen) consumption.

	Median number of tablets consumed	Paracetamol users	
		*n*	%
Diclofenac	4.0^1,AB^ (0.0, 23.0)	26	56.5
Wobenzym	0.5^A^ (0.0, 7.0)	26	50.0
Placebo	10.0^B^ (2.0, 22.0)	33	63.5

^1^All values presented as medians (95% confidence interval).

^A,B^Treatment groups with different superscripts differ within the same column (*P* < 0.05).

**Table 5:** Adverse events resulting in discontinuation of treatment.

Treatment group	Gender	Adverse events
Diclofenac	F	Nausea
	F	Diarrhea
	F	Acute leukocytosis
	F	Eczema
	F	Stomach pain
	M	Edema

Wobenzym	F	Heartburn
	M	Stomach pain
	M	Back pain
	M	Stomach pain accompanied by gastroesophageal reflux and nausea

Placebo	F	Nausea
	F	Nausea
	F	Edema of legs
	M	Flatulence and diarrhea
	M	Abnormal laboratory results
	M	Stomach pain and episodes of vertigo

## References

[1] Lawrence R. C., Felson D. T., Helmick C. G. (2008). Estimates of the prevalence of arthritis and other rheumatic conditions in the United States. Part II. *Arthritis and Rheumatism*.

[2] Felson D. T., Naimark A., Anderson J., Kazis L., Castelli W., Meenan R. F. (1987). The prevalence of knee osteoarthritis in the elderly. The Framingham Osteoarthritis Study. *Arthritis & Rheumatism*.

[3] Abramson S. B., Attur M. (2009). Developments in the scientific understanding of osteoarthritis. *Arthritis Research & Therapy*.

[4] Felson D. T. (2009). Developments in the clinical understanding of osteoarthritis. *Arthritis Research and Therapy*.

[5] Altman R. D. (2010). Early management of osteoarthritis. *The American Journal of Managed Care*.

[6] McGettigan P., Henry D. (2011). Cardiovascular risk with non-steroidal anti-inflammatory drugs: systematic review of population-based controlled observational studies. *PLoS Medicine*.

[7] Akhtar N. M., Naseer R., Farooqi A. Z., Aziz W., Nazir M. (2004). Oral enzyme combination versus diclofenac in the treatment of osteoarthritis of the knee—a double-blind prospective randomized study. *Clinical Rheumatology*.

[8] Singer F., Oberleitner H. (1996). Osteoarthritis of the knee—effectiveness of enzymes vs diclofenac in acute phase of osteoarthritis of the knee—a randomized, double blind controlled study. *Wiener Medizinische Wochenschrift*.

[9] Singer F., Singer C., Oberleitner H. (2001). Phlogenzym versus diclofenac in the treatment of activated osteoarthritis of the knee. A double-blind prospective randomized study. *International Journal of Immunotherapy*.

[10] Klein G., Kullich W., Schnitker J., Schwann H. (2006). Efficacy and tolerance of an oral enzyme combination in painful osteoartritis of the hip. A double-Blind, randomised study comparing oral enzymes with non-steroidal anti-inflammatory drugs. *Clinical and Experimental Rheumatology*.

[11] Klein G., Kullich W. (2000). Short-term treatment of painful osteoarthritis of the knee with oral enzymes. *Clinical Drug Investigation*.

[12] Lequesne M. G. (1997). The algofunctional indices for hip and knee osteoarthritis. *Journal of Rheumatology*.

[13] Bellamy N., Buchanan W. W., Goldsmith C. H., Campbell J., Stitt L. W. (1988). Validation study of WOMAC: a health status instrument for measuring clinically important patient relevant outcomes to antirheumatic drug therapy in patients with osteoarthritis of the hip or knee. *Journal of Rheumatology*.

[14] Reitzenstein J. E., Yamamoto L. G., Mavoori H. (2010). Similar erythrocyte sedimentation rate and C-reactive protein sensitivities at the onset of septic arthritis, osteomyelitis, acute rheumatic fever. *Pediatric Reports*.

[15] Bluman A. G. (2004). *Elementary Statistics. A Step-by-Step Approach*.

[16] Mazourov V. I., Lila A. M., Klimko N. N., Raimuev K. V., Makulova T. G. (1997). The efficacy of systemic enzyme therapy in the treatment of rheumatoid arthritis. *International Journal of Immunotherapy*.

[17] Friedrich F. (1993). Therapie der chronischen Adnexitis: Diclofenac oder Enzyme? Randomisierte doppelblinde Parallelgruppe gegen Diclofenac. *Der Allgemeinarzt*.

[18] Lackovic V., Rovensky J., Horvathová M., Malis F. (1997). Interferon production in whole blood cultures from volunteers and rheumatoid arthritis patients after medication with oral enzymes. *International Journal of Immunotherapy*.

[19] Kovalenko V. B., Shuba N. M., Golovatskiy I. V., Bortkevich O. P., Yasinskaya V. A. (2001). Estimation of efficacy of basic therapy of rheumatoid arthritis on the basis of systemic enzyme therapy: results of five-year monitoring. *International Journal of Immunotherapy*.

[20] Klein G., Kullich W. (1999). Pain reduction in rheumatic diseases by oral therapy with enzymes. *Wiener Medizinische Wochenschrift*.

[21] Szczurko O., Cooley K., Mills E. J., Zhou Q., Perri D., Seely D. (2009). Naturopathic treatment of rotator cuff tendinitis among Canadian postal workers: a randomized controlled trial. *Arthritis Care and Research*.

[22] Leipner J., Iten F., Saller R. (2001). Therapy with proteolytic enzymes in rheumatic disorders. *BioDrugs*.

[23] Wittenborg A., Bock P. R., Hanisch J., Saller R., Schneider B. (2000). Comparative epidemiological study in patients with rheumatic diseases illustrated in an example of a treatment with non-steroidal anti-inflammatory drugs versus an oral enzyme combination. *Drug Research*.

[24] Malovic P. (2008). Enzymoterapia futbaloyych urazov. *Časopis Medicina Sportiva Bohemica & Slovaca*.

[25] Wörschhauser S., Zuschlag J. M. (1991). Prophylaxe der weichteilverletzungen bei kontakt sportarten. *Der Allgemeinarzt*.

[26] Doenicke A., Hoernecke R. (1993). Wirksame behandlung von traumen mit schwellungen und/oder hämatom im eishockesport durch enzymtherapie. *Deutsche Zeitschrift für Sportmedizin*.

[27] Rahn H. D. (1995). Verkürzte heilzeiten bei systemischer therapie mit hydrolytischen enzymen. *Deutsche Zeitschrift für Sportmedizin*.

[28] Enck P., Bingel U., Schedlowski M., Rief W. (2013). The placebo response in medicine: minimize, maximize or personalize?. *Nature Reviews Drug Discovery*.

[29] Zhang W., Robertson J., Jones A. C., Dieppe P. A., Doherty M. (2008). The placebo effect and its determinants in osteoarthritis: meta-analysis of randomised controlled trials. *Annals of the Rheumatic Diseases*.

[30] Brien S., Lewith G., Walker A. F., Middleton R., Prescott P., Bundy R. (2006). Bromelain as an adjunctive treatment for moderate-to-severe osteoarthritis of the knee: a randomized placebo-controlled pilot study. *QJM*.

[31] Paź A., Zaborski J., Kruszewska-Ozimowska J., Członkowski A., Członkowska A. (2002). Analysis of peripheral blood leukocyte immunophenotype during oral hydrolytic enzyme treatment of multiple sclerosis patients. *International Journal of Immunotherapy*.

[32] Klein G., Kullich W., Schwann H. (1995). Double-blind trial of an enzyme therapy versus oral gold in rheumatoid arthritis. *Clinicum*.

[33] Kovalenko V. B., Shuba N. M., Golovatskiy I. V., Bortkevich O. P., Yasinskaya V. A. (2001). Estimation o f efficacy of basic therapy of rheumatoid arthritis on the basis of systemic enzyme therapy: results of five-year monitoring. *International Journal of Immunotherapy*.

