# Supplementary material for: The Safety and Efficacy of an Enzyme Combination in Managing Knee Osteoarthritis Pain in Adults: A Randomized, Double-Blind, Placebo-Controlled Trial
Source: Arthritis. 2015 Jan 31;2015:251521. doi: 10.1155/2015/251521 (PMC4329848; doi:10.1155/2015/251521)
Supplement: Supplementary file 1 — Subjects were administered diclofenac, Wobenzym®, or placebo for 12 weeks. No changes in vital signs or blood chemistries were observed. [file 251521.f1.docx]

Arthritis

**The safety and efficacy of an enzyme combination in managing knee osteoarthritis pain in adults: a randomized, double-blind, placebo-controlled trial.**

Wolfgang W. Bolten MD, Michael J. Glade PhD, Sonja Raum, Barry W. Ritz PhD

Corresponding Author:
Barry W. Ritz

Atrium Innovations, Chadds Ford, PA, USA
Phone: (610) 361-2800 x 15

Fax: (610) 361-6168
[br@atrium-innovations.com](mailto:br@atrium-innovations.com)

Description: Subjects were administered diclofenac, Wobenzym^®^, or placebo for 12 weeks. No changes in vital signs or blood chemistries were observed.

Table- Supplemental Data. Vital signs, clinical chemistry, and hematology.

|  | Diclofenac | | Wobenzym^®^ | | Placebo | |
| --- | --- | --- | --- | --- | --- | --- |
|  | Week 0 | Week12 | Week 0 | Week12 | Week 0 | Week12 |
| **Vital signs:** | | | | | | |
| **Resting systolic blood pressure (mm Hg)** | | | | | | |
| median | 140 | 140 | 140 | 140 | 140 | 140 |
| 95% CI | 130-145 | 135-150 | 130-145 | 135-149 | 130-150 | 130-140 |
| **Resting diastolic blood pressure (mm Hg)** | | | | | | |
| median | 80 | 80.5 | 80.5 | 82.5 | 80 | 80 |
| 95% CI | 80-86 | 80-90 | 80-85 | 80-85 | 78-90 | 80-88 |
| **Resting heart rate (bpm)** | | | | | | |
| median | 70.5 | 76 | 70 | 72 | 72 | 76 |
| 95% CI | 68-77 | 70-82 | 68-80 | 68-84 | 68-76 | 70-82 |
| **Resting body temperature (°C)** | | | | | | |
| median | 36.8 | 36.8 | 36.8 | 37.0 | 36.8 | 36.3 |
| 95% CI | 36.6-37.0 | 36.4-37.0 | 36.4-36.9 | 36.7-37.0 | 36.5-37.0 | 36.6-37.0 |
| **Concentrations in serum:** | | | | | | |
| **total protein (g/L)** | | | | | | |
| median | 70 | 69 | 69 | 69 | 70 | 69 |
| 95% CI | 67-72 | 67-73 | 68-72 | 67-73 | 68-72 | 68-71 |
| **albumin (g/l)** | | | | | | |
| median | 39 | 40 | 39 | 40 | 40 | 40 |
| 95% CI | 37-41 | 38-42 | 37-42 | 38-42 | 39-41 | 38-41 |
| **urea-nitrogen (mg/dL)** | | | | | | |
| median | 15.4 | 16.4 | 15.4 | 15.4 | 14.3 | 15.2 |
| 95% CI | 14.0-17.8 | 14.0-13.2 | 13.6-17.3 | 14.0-17.8 | 13.6-16.8 | 13.1-16.8 |
| **creatinine (mg/dL)** | | | | | | |
| median | 0.92 | 0.90 | 0.90 | 0.89 | 0.87 | 0.82 |
| 95% CI | 0.82-1.00 | 0.77-0.99 | 0.83-0.98 | 0.89-1.01 | 0.76-0.97 | 0.74-0.91 |
| **total bilirubin (mg/dL)** | | | | | | |
| median | 0.42 | 0.45 | 0.38 | 0.37 | 0.45 | 0.44 |
| 95% CI | 0.32-0.54 | 0.34-0.62 | 0.27-0.48 | 0.30-0.48 | 0.34-0.54 | 0.38-0.56 |
| **hemoglobin (g/dL)** | | | | | | |
| median | 14.1 | 13.8 | 14.2 | 13.8 | 14.1 | 14.0 |
| 95% CI | 13.2-14.6 | 13.2-14.1 | 13.0-14.5 | 13.3-14.4 | 13.7-14.6 | 13.3-14.7 |
| **glucose (mg/dL)** | | | | | | |
| median | 99 | 102 | 102 | 102 | 95 | 95 |
| 95% CI | 92-104 | 89-129 | 93-110 | 94-116 | 89-113 | 89-114 |
| **sodium (mmol/L)** | | | | | | |
| median | 141 | 140 | 140 | 140 | 140 | 140 |
| 95% CI | 139-142 | 139-141 | 139-141 | 137-141 | 139-141 | 138-141 |
| **potassium (mmol.L)** | | | | | | |
| median | 4.3 | 4.5 | 4.4 | 4.5 | 4.4 | 4.4 |
| 95% CI | 4.2-4.7 | 4.4-4.8 | 4.2-4.6 | 4.1-4.7 | 4.3-4.5 | 4.1-4.7 |
| **chloride (mmol/L)** | | | | | | |
| median | 103 | 103 | 103 | 102 | 101 | 100 |
| 95% CI | 101-107 | 100-105 | 100-105 | 99-103 | 100-104 | 97-102 |
| **calcium (mmol/L)** | | | | | | |
| median | 2.36 | 2.36 | 2.43 | 2.39 | 2.41 | 2.40 |
| 95% CI | 2.32-2.43 | 2.32-2.43 | 2.35-2.46 | 2.35-2.44 | 2.35-2.45 | 2.36-2.43 |
| **Activities in serum:** | | | | | | |
| **alkaline phosphatase (U/L)** | | | | | | |
| median | 95 | 93 | 97 | 97 | 100 | 101 |
| 95% CI | 81-104 | 81-113 | 87-112 | 86-121 | 86-112 | 90-119 |
| **glutamic-oxaloacetic transaminase (SGOT; ASAT) (U/L)** | | | | | | |
| median | 8 | 10 | 9 | 10 | 9 | 9 |
| 95% CI | 7-10 | 8-13 | 8-10 | 8-11 | 8-10 | 8-10 |
| **glutamic/glutamate pyruvic transaminase (SGPT; ALAT) (U/L)** | | | | | | |
| median | 9 | 11 | 10 | 11 | 11 | 11 |
| 95% CI | 8-11 | 9-16 | 8-13 | 8-12 | 9-13 | 9-13 |
| **γ-glutamyl transpeptidase (γ-GT; GGT) (U/L)** | | | | | | |
| median | 10 | 12 | 11 | 13 | 13 | 13 |
| 95% CI | 9-13 | 10-21 | 9-15 | 9-20 | 10-18 | 10-19 |
| **Cell counts:** | | | | | | |
| **white blood cell count (cells/nL)** | | | | | | |
| median | 6.0 | 6.4 | 6.7 | 6.9 | 6.5 | 6.9 |
| 95% CI | 5.3-7.3 | 5.7-7.2 | 6.1-7.8 | 6.0-7.9 | 5.7-7.2 | 5.8-7.9 |
| **red blood cell count (cells/nL)** | | | | | | |
| median | 4.69 | 4.53 | 4.61 | 4.55 | 4.53 | 4.58 |
| 95% CI | 4.39-4.83 | 4.39-4.70 | 4.33-4.89 | 4.39-4.81 | 4.44-4.71 | 4.41-4.80 |
| hematocrit (%) | | | | | | |
| median | 43.2 | 42.6 | 43.4 | 42.9 | 43.4 | 43.6 |
| 95% CI | 41.8-45.7 | 40.2-43.3 | 41.6-45.1 | 41.5-44.2 | 41.9-44.7 | 41.6-45.1 |
| thrombocytes (cells/nL) | | | | | | |
| median | 257 | 243 | 274 | 278 | 253 | 246 |
| 95% CI | 225-284 | 212-283 | 246-315 | 239-307 | 227-274 | 230-271 |
| thromboplastin time (%) | | | | | | |
| median | 100 | 102 | 99 | 102 | 99 | 101 |
| 95% CI | 95-103 | 97-108 | 94-102 | 98-107 | 94-105 | 97-109 |
| partial thromboplastin time (sec) | | | | | | |
| median | 33.8 | 33.9 | 34.1 | 33.4 | 33.8 | 34.6 |
| 95% CI | 32.8-35.5 | 32.7-35.6 | 31.8-36.6 | 31.8-36.2 | 32.5-35.9 | 32.6-36.0 |
